# The Effect of Hollow Polymer Microspheres on the Pore Structure and Frost Resistance of Cement Mortar

**DOI:** 10.3390/ma18071644

**Published:** 2025-04-03

**Authors:** Lihui Li, Jianrui Ji, Lingfeng Yu, Zhihui Luo, Panpan Zhang, Kaimin Niu

**Affiliations:** 1 School of Civil Engineering, Chongqing Jiaotong University, Chongqing 400000, China; lilihui0451@163.com (L.L.); 18293198316@163.com (Z.L.); zhangpanpan648@126.com (P.Z.); 2 Institute of Highway, Ministry of Transport, Beijing 100088, China; 3 School of Civil Engineering and Transportation, South China University of Technology, Guangzhou 850015, China; 202320107962@mail.edu.cn

**Keywords:** air-entrained mortar, WEA, pore structure parameters, spacing coefficient, frost resistance

## Abstract

This study elucidates the effects of total air content, pore size distribution, and bubble spacing coefficient on mortar frost resistance. It presents a systematic comparison between mortars obtained with different porous structures. Conventional porous mortars and mortars with controlled porosity were prepared using air-entraining agents (SJ2) and hollow polymer microspheres with a controllable particle size (WEA, 20–80 μm), respectively. The study shows that WEA can construct uniformly sized and regularly shaped pores in mortar and can introduce controllable and stable pore sizes compared with SJ2, which are almost independent of mixing, molding, hydration, and hardening factors. Under equivalent air content, WEA mortar exhibits superior mechanical properties and frost resistance compared to SJ2 mortar, showing a negative correlation with WEA particle size. The frost resistance of WEA (40 μm) mortar with 1% volume content is comparable to that of SJ2 air-entrained mortar with 4% air content; i.e., compared to an SJ2 air-entraining agent, to achieve the same frost resistance level, WEA mortar has lower air content and a larger bubble spacing coefficient.

## 1. Introduction

The distinctive high-altitude, low-pressure environment of the Tibetan Plateau, along with frequent alternation between positive and negative temperatures, has resulted in significant frost–thaw damage to the concrete structures of highway infrastructure [1]. Therefore, the need to enhance the freezing durability of concrete in the plateau region should not be ignored. Currently, the main means of improving the frost resistance of concrete is to introduce tiny pores through an air-entraining agent to achieve the purpose of buffering the damage of freezing and thawing stress, and most of the existing research focuses on the effects of the air-entraining agent used [2,3], the air-entraining agent content [4,5], the pore structure parameters [6,7], mineral admixtures [8,9], the environmental conditions, etc. [10,11,12], on the concrete’s frost resistance, and there are few reports on the relationship between the frost resistance of concrete and the type of air-entraining agent used, the way bubbles cluster, the size of bubble clusters, the pore morphology characteristics, etc. There are few reports on the relationship between factors such as the type and mode of air entrainment, pore size, pore shape characteristics, and the frost resistance of concrete. Liu Xu [13] and Li Lihui [14] showed that the air-entraining pores of concrete under the low air pressure in the plateau are coarsened, and the bubbles are easier to burst, which leads to an increase in the average radius of the pores and the coefficient of the spacing of the bubbles and influences the structure of the pores of the concrete and its frost resistance. Yang Qianrong [15] and Zhang Jinxi et al. [16] found that it is not easy to control the parameters of the bubbles of traditional air-entrained concrete during mixing and transportation and under high-frequency vibration and pounding construction, which results in the escape of bubbles from the concrete and even their bursting and undermines the frost resistance of concrete. It is well known that the key factors affecting the freezing resistance of concrete utilizing air-entraining are air content, pore size, and the bubble spacing coefficient [17]. Li Hao [18] investigated the law influencing cement hydration and pore characteristics in concrete by preparing a type of calcium acrylate microsphere with very strong water retention. It was shown that when its dosage was 1.5%, it could significantly enhance the late strength of the concrete, reduce the proportion of harmful pores inside the concrete, improve the pore structure, and improve the freezing resistance of the concrete. Ong et al. [19] used a type of solid bead with a particle size of only a few micrometers to study the effect of its incorporation into mortar on the frost resistance of the mortar and found that the mortar with a particle size of 6 μm and a dosage of 2% beads had a better resistance to freezing and thawing strains, and it is believed that the beads and the mortar, due to the difference in the coefficients of thermal expansion, would form pores in the transition zone of their interfaces in order to resist the freezing and expanding stresses.

Existing research predominantly emphasizes the optimization of individual variables, such as types of air-entraining agents and environmental parameters, while neglecting the precise regulation of pore systems across different scales. This oversight is particularly pronounced with regard to the effects of pore size distribution heterogeneity and morphological irregularity on the frost resistance of concrete, which represents a theoretical blind spot in the design of frost-resistant concrete for high-altitude environments. Therefore, in this paper, a hollow polymer microsphere (WEA) material is used to introduce specific-sized pores into mortar, according to the introduction of hollow polymer microspheres with different particle sizes to realize the introduction of pores with different target size intervals into the mortar system. By comparing it with conventional air-entraining agents (SJ2) for air-entrained mortar, we discuss the differences in the pore structure parameters of the pore groups with different target sizes and those of the traditional large pore groups. The relationship between different stomatal structure parameters and the frost resistance of mortar is investigated at the macro- and microscopic levels. This is of great theoretical significance and practical value for the selection of the type of air-entraining agent in air-entrained concrete under the environment of the Tibetan Plateau, as well as for the quantification of the magnitude of the effect of bubble cluster size on the frost resistance of air-entrained concrete.

## 2. Materials and Methods

### 2.1. Raw Materials

**Figure 1:**
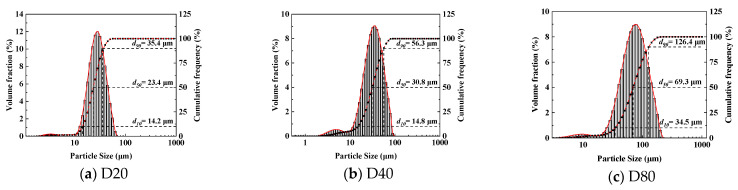
Laser particle size test results of WEA.

P.I 42.5 standard cement produced by Fushun Cement Co., Ltd. (Fushun, China) was used in the cement mortar test, and the chemical composition and physical properties of the standard cement are shown in Table 1 and Table 2. The sand used in the test was China ISO standard sand produced by Xiamen Aisio Standard Sand Co. (Xiamen, China). The water used for mixing was municipal tap water. The traditional air-entraining agent was SJ-2 (hereinafter referred to as SJ2), and the hollow polymer microspheres were EXPANCEL WEA series products (hereinafter referred to as WEA) produced by Nolion, which are a kind of microsphere made of thermoplastic resin shell and filled with alkane gas, and the outer surface of the shell is wrapped with a layer of water film, which can be effectively dispersed in the cement paste. The relevant parameters of the material are shown in Table 3. The particle size distribution was tested using a BT9300S laser particle size distribution meter manufactured in Shanghai, China [20], and the particle size distribution is shown in Figure 1.

### 2.2. Test Ratios

In this study, the water-to-cement ratio of the benchmark cement mortar was 0.45, and the cement-to-sand ratio was 0.5. The air content of the WEA air-entraining mortar was determined via isovolumetric substitution based on the benchmark group’s ratio (2% air content). Bulk density measurements were conducted at varying air contents, and the air content of the SJ2 air-entraining mortar was determined through interpolation based on the relationship between the air content and the bulk density. The test measured SJ2 gas content of 3%, 6%, 9%, corresponding to dosages of 1.8‱, 3.9‱, and 7.8‱. The WEA air-entraining mortar components of the ratio are shown in Table 4, in which D20, D40, and D80 represent the WEA particle size specifications of 20 μm, 40 μm, and 80 μm, respectively.

### 2.3. Test Methods

Air content: The air content of the mortar after hardening was back-calculated for each set of hardened mortar specimens using Equation (1) based on the apparent density after hardening [9,10]:

(1) $$A=\frac{\rho_{0}-\rho }{\rho_{0}}\times 100\%$$

where *A* is the air content of cement mortar after hardening (%); $\rho_{0}$ is the skeleton density of cement mortar (kg/m^3^); and ρ is the apparent density of cement mortar (kg/m^3^).

Mechanical properties and micro-morphological analysis: The compressive strength of the mortar was tested at 3, 14, and 28 days of curing, using a WDW-type electric servo press manufactured in the United States, according to GB/T 17671-2021 [21]. The morphologies of the mortars were observed by using an SEM (JSM-5600LV, Nippon Electro-Optics, Inc., Tokyo, Japan). Before testing, all samples were crushed into small pieces and then dried to constant weight in an oven at 60 °C [22].

Residual strain measurement: Strain gauges with a resistance value of 350 Ω were selected. For each group, three 40 mm × 40 mm × 160 mm mortar specimens were prepared and subjected to standard curing for 24 days. Subsequently, the specimens were immersed in (20 ± 2) °C water for 3 days to achieve saturation, bonded with H-610 strain gauge adhesive, waterproofed using silica gel sealant, and transferred to a dedicated specimen box. The specimens were then placed in the specimen box for 24-h water-saturation pretreatment. After pretreatment, the water was drained from the specimen box, and the box was sealed with a signal line. The sealed specimen box was placed in a freeze–thaw chamber, maintaining an internal temperature range of −18 °C to 5 °C. Each freeze–thaw cycle lasted 4 h, consisting of a 2.5-h cooling phase (5 °C to −18 °C) and a 1.5-h warming phase (−18 °C to 5 °C). Finally, the signal line was connected to a data collector and computer for real-time strain monitoring during the freeze–thaw cycles.

Mercury intrusion porosimetry: The pore size was tested using the United States Quantachrome production model PM60GT−17 pressed mercury porosimeter, which has a test range exceeding 3 nm. For the test, broken mortar specimens were trimmed into spherical particles with a diameter of 2 to 5 mm using a tool clamp. The trimmed specimens were soaked in anhydrous ethanol for 24 h, after which the ethanol was replaced with fresh anhydrous ethanol, and the specimens were soaked for an additional 24 h to halt the hydration reaction. Prior to the mercury intrusion test, the specimens were removed and placed in an oven to dry at 90 °C for 12 h. Once cooled to room temperature, the mercury pressure test was conducted.

Pore structure test of hardened mortar: The pore structure of hardened cement mortar was analyzed using the Air-rapid 457 Hardened Concrete Pore Analyzer, made in Germany, which has an accuracy of 10 µm. This analysis focused on key pore structure parameters, including air content and bubble spacing coefficient. Additionally, the porosity parameters of hardened concrete were evaluated according to the linear conductor method specified in ASTM C457 [22].

## 3. Results and Discussion

### 3.1. Gas Content

**Figure 2:**
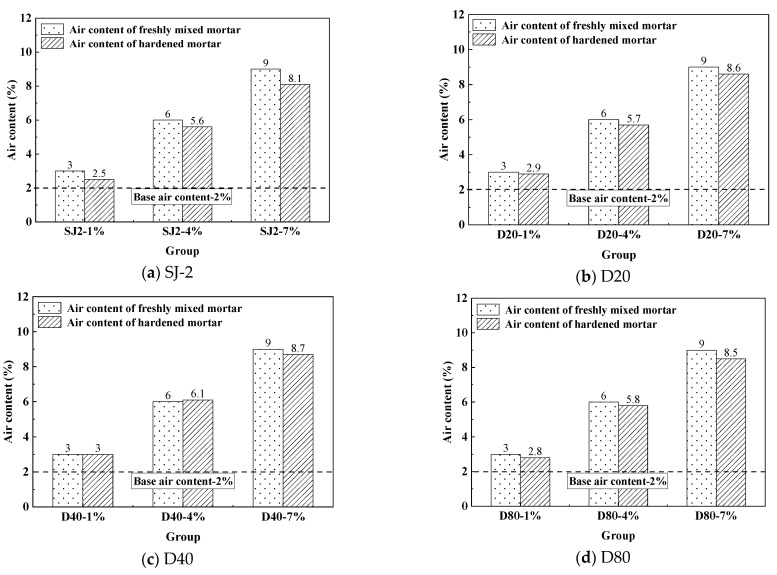
Air content of the mortar.

By incorporating WEA, the volume of the mortar medium can be altered to design the target air content (as shown in Table 4). The gas content of the traditional SJ2 air-entrained mortar can be established by referring to the WEA gas content to determine the gas content of each group of fresh mortar and hardened mortar, as shown in Figure 2.

As illustrated in Figure 2, both the SJ2 air-entraining agent and the polymer-based hollow microsphere WEA agent significantly increase mortar air content, though the hardened mortar’s air content is lower than that of the fresh mix. Comparing pre- and post-hardening air content, the SJ2-modified mortar exhibited a 17% reduction, whereas the WEA-modified mortar showed only a 3–7% decline, indicating a markedly lower rate of air content loss. This demonstrates that WEA-introduced pores exhibit greater stability during hardening compared to SJ2’s liquid film bubbles, retaining air content more effectively. This enhanced performance stems from WEA’s structure: gas-filled microspheres possess a 100 nm thick thermoplastic shell, which provides superior mechanical strength and dimensional stability during mixing, molding, and curing compared to the fragile liquid film bubbles formed by conventional agents like SJ2.

### 3.2. Compressive Strength

**Figure 3:**
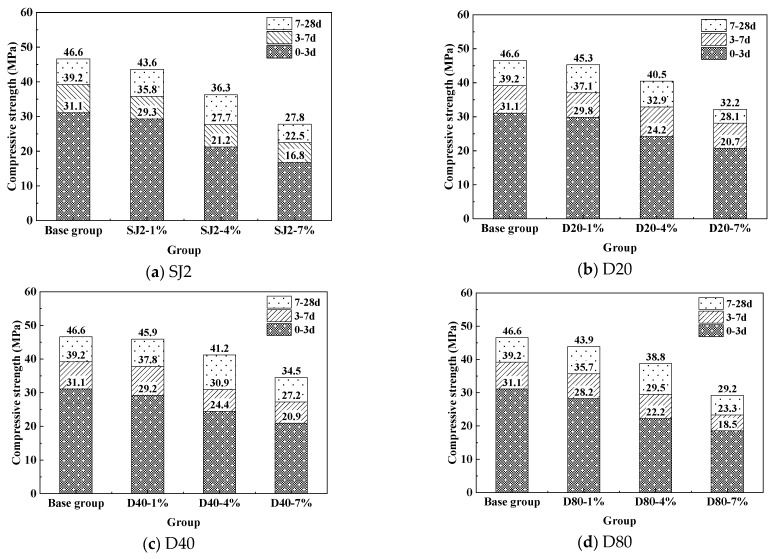
Effect of air content on the compressive strength of mortar.

The compressive strengths of conventional SJ2 air-entraining mortar and WEA air-entraining mortar at all ages are shown in Figure 3.

As can be seen from Figure 3, the compressive strength of each group of mortar decreases with the increase in introduced gas content. When the gas content is the same, the compressive strength of the WEA-entrained mortar is higher than that of the SJ2-entrained mortar; when the gas content is 9%, and the compressive strength of the benchmark group compared to the 28d compressive strength of the SJ2-entrained mortar decreased by 18.8Mpa, a decrease of 40.3%, while the compressive strength of the WEA-entrained mortar mixed with three different particle sizes was less than that of the SJ2 mortar, with the smallest decrease of 26.0%. The reason is due to the fact that the WEA material itself has a thermoplastic shell, filled with alkane gas, and the spherical shell structure of the WEA compared with the traditional liquid film bubbles of the SJ2 has better strength and stability, the size is more uniform, making it better able to fill the gap between the cement and aggregate, and then the compressive strength is higher. Comparing the WEA with different particle sizes, the compressive strength of mortar decreases with the increase in the introduced particle size when the same air content is introduced, indicating that the larger the introduced pore size, the more unfavorable it is to the compressive strength of the mortar.

### 3.3. Solid Air-Entraining Mortar Pore Structure

#### 3.3.1. MIP

**Figure 4:**
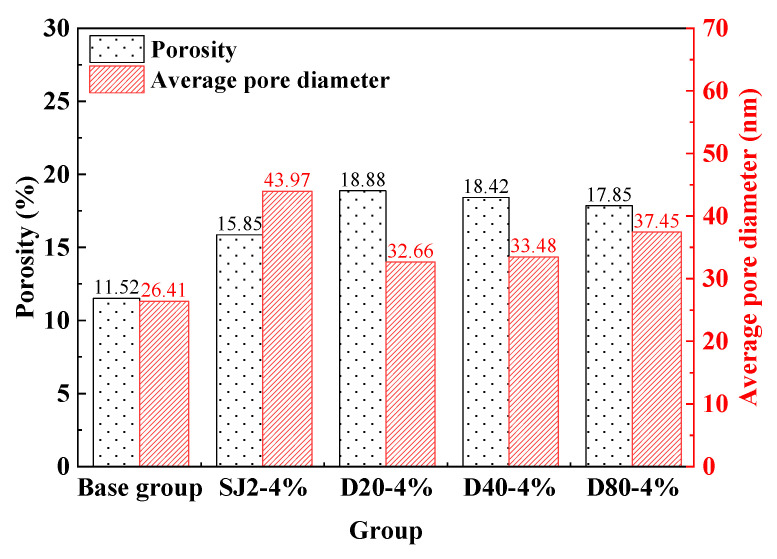
The total porosity and average pore size of each group of air-entrained mortars.

To evaluate the pore distribution in mortars with varying air-entraining methods and pore size ranges, five groups—benchmark (non-air-entrained), SJ2-4%, D20-4%, D40-4%, and D80-4%—were prepared and cured for 28 days under standard conditions. Porosity and average pore diameter were quantified using mercury intrusion porosimetry (MIP), as summarized in Figure 4. As can be seen in Figure 4, air-entrained mortars exhibited higher total porosity and larger average pore diameters compared to the non-air-entrained benchmark. However, WEA-modified mortars showed lower average pore diameters than SJ2 mortars. As reported in prior studies [23], air-entrained mortars typically exhibit pore sizes concentrated in the 20–200 μm range, though larger pores (>200 μm), classified as harmful, may also form. WEA introduces smaller, more uniformly distributed and structurally stable pores, resulting in higher total porosity and reduced average pore size at equivalent air content compared to conventional air-entraining agents like SJ2.

**Figure 5:**
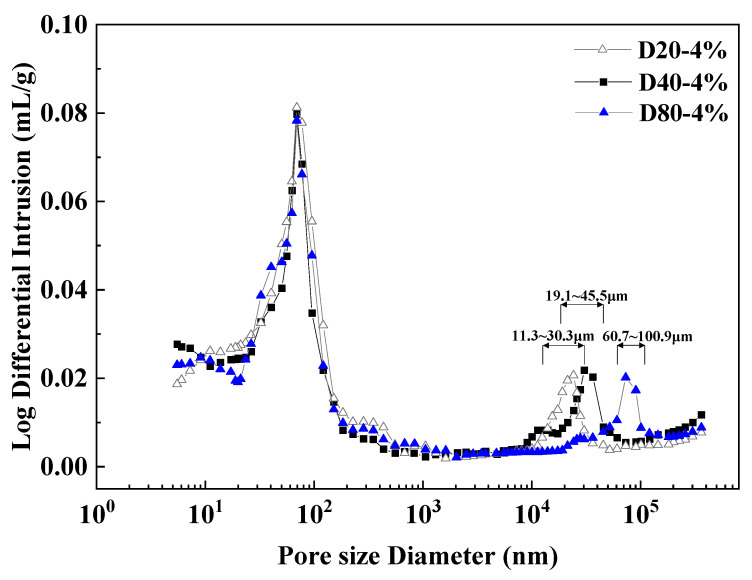
Pore size distribution of WEA mortar with different particle sizes.

In order to study the characteristics of the pore structure parameters of WEA air-entraining mortar with different particle sizes after hardening, the specimens of D20-4% air-entraining mortar, D40-4% air-entraining mortar, and D80-4% air-entraining mortar were selected to conduct the mercury pressure test after 28 days of standard curing, and the results are shown in Figure 5. It can be seen from Figure 5 that there are two peaks in the pore size distribution curves of the different sizes of WEA mortar, and the left peak corresponds to the range of the pore sizes of 20~100 nm. The difference in the pore size distribution curves is not significant. The right peaks correspond to pore size ranges of 11.3~30.3 μm, 19.1~45.5 μm, and 60.7~100.9 μm for D20-4% mortar, D40-4% air-entraining mortar, and D80-4% air-entraining mortar, respectively. The pore sizes corresponding to the peaks of the differential mercury feed curves in a certain pore size range are the most abundant. As can be seen from the pore size distribution graphs, the most abundant pore sizes corresponding to the right peaks of the three differential mercury feed curves are 21.3 μm, 30.3 μm and 73.0 μm, respectively, which are smaller than the WEA material parameters as a whole, which may be attributed to the volume reduction of the sphere due to the environmental factors before mixing it into the mortar; furthermore, the surface of the cement particles generates C-S-H and other hydration products to fill the pores and this may also lead to a reduction in differential mercury intake.

#### 3.3.2. SEM

**Figure 6:**
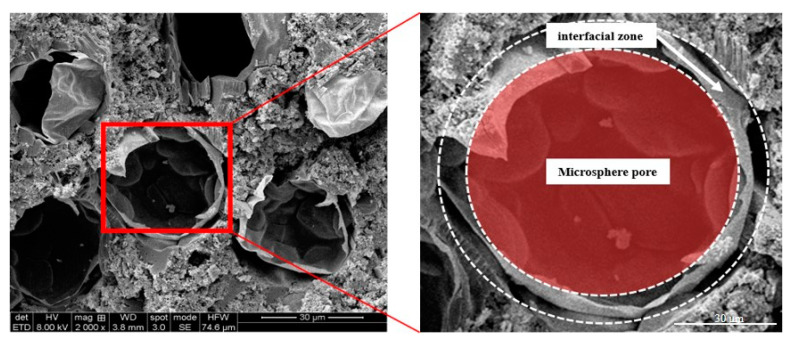
SEM (D40) image of WEA mortar.

Scanning electron microscopy (SEM) was employed to analyze the microscopic morphology of WEA and the pore structure within the hardened mortar, with the aim of elucidating its anti-freezing mechanism. WEA-D40 specimens, cured under standard conditions for 28 days, were selected for SEM analysis; the results are presented in Figure 6. As shown in Figure 6, the WEA spheres feature a shell composed of organic thermoplastic resin, encapsulated by a water film. This film is progressively depleted during hydration, leaving an organic layer that fails to bond effectively with the cementitious matrix, thereby creating a distinct interfacial transition zone. During prolonged curing, the WEA spheres undergo progressive shrinkage and fracture, ultimately yielding uniformly distributed pores. The higher thermal expansion coefficient of WEA relative to the surrounding mortar matrix renders it susceptible to volumetric instability and interfacial debonding under temperature fluctuations. With the increase in curing age, the degree of hydration in the mortar increases, and the accumulation of hydration products, especially the needle-like silicate and carbonate mineral crystals, also leads to the rupture and shrinkage of WEA microspheres.

### 3.4. Frost Resistance

#### 3.4.1. Residual Strain

**Figure 7:**
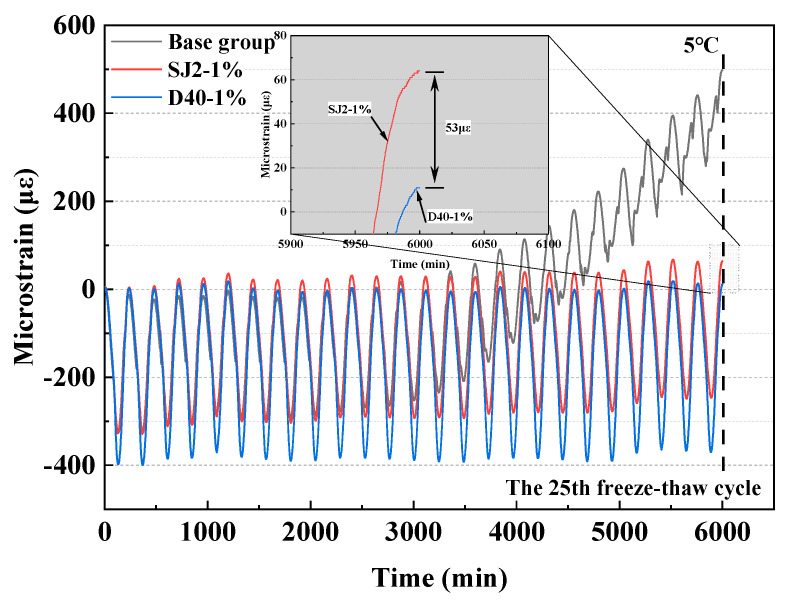
Strain plot of the 25 freeze–thaw cycles over time.

In order to discuss the influence of different air-entraining methods on the frost resistance of mortar, this paper analyzes the relationship between strain versus time during the first 25 freeze–thaw cycles of mortar specimens of the three groups of the baseline group (un-entraining), SJ2 with 1% air-entraining, and D40, and the results are shown in Figure 7.

Based on the principle of freeze–thaw damage, the compressive stress produced by the matrix material in the three macroscopic groups of freeze–thaw cycle tests plays a dominant role, resulting in the mortar specimen showing a shrinkage trend with the decrease in temperature [24]. From Figure 7, it can be seen that during the first 25 freeze–thaw cycles, the baseline group is the first to undergo obvious deformation compared with other air-entraining groups, and obvious irreversible deformation has already occurred after only 15 freeze–thaw cycles, and the microstrain value is as high as 498 με after 25 freeze–thaw cycles, which indicates that un-entraining mortar is extremely prone to freeze–thaw damage during the freeze–thaw cycles. Comparing the deformation characteristics of SJ2-1% and D40-1% mortars with 1% air content, it can be seen that the microstrain of the air-entraining mortar doped with SJ2 reaches 53 με after 25 freezing and thawing cycles, whereas the mortar doped with WEA does not have any obvious residual strain on the whole. This indicates that the WEA mortar with the same air content has better resistance to freezing stress than the SJ2 mortar, which is due to the introduction of a regular spherical pore structure in the mortar by WEA, which can maximally offset the tensile stress generated by the pore water freezing, slow down the increase in the residual strain of the mortar, and then improve the frost resistance of the mortar.

**Figure 8:**
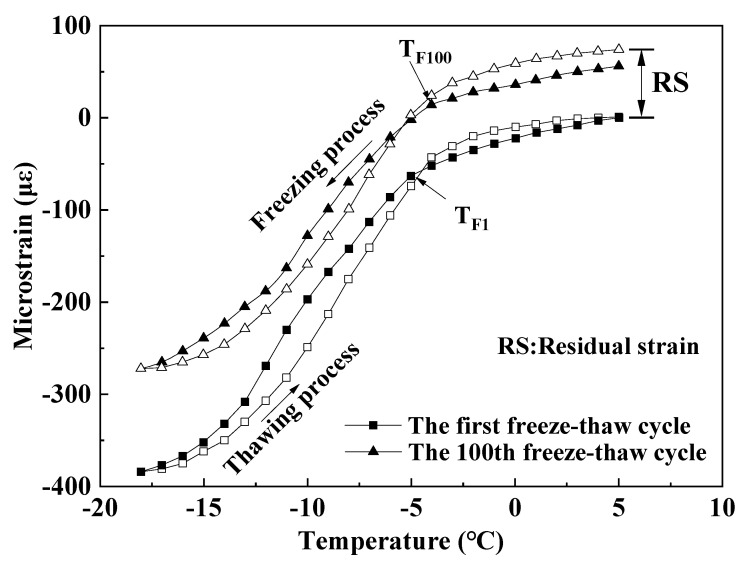
Diagram of the strain of D40-1% mortar as a function of temperature in the 1st and 100th freeze–thaw cycles.

In order to analyze the change rule of the strain curve of WEA-doped mortar after a certain number of freeze–thaw cycles, the 1st and 100th freeze–thaw cycles of D40-1% microstrain with temperature changes were selected, and the results are shown in Figure 8.

From Figure 8, it can be seen that the overall upward shift of the microstrain versus temperature curve of the mortar after 100 freeze–thaw cycles indicates that irreversible residual deformation of the mortar is generated. Although the WEA-doped mortar can introduce a large number of uniform micropores to dissipate the freeze–thaw stresses, with the increase in freeze–thaw frequency and the thermal expansion and contraction deformation of the mortar matrix itself, the accumulation of residual strain in the mortar increases, and ultimately, the material will be freeze–thaw damaged. In addition, Figure 8 also shows that the strain paths of mortar specimens during freezing and thawing do not overlap but instead form an obvious hysteresis loop, which is due to the existence of the “ink bottle effect” [25], and the experimental observation of the strain curve of each freeze–thaw cycle shows that there is an obvious strain inflection point in the freezing stage, and its corresponding temperature is called the characteristic freezing temperature T_F_ [26]. This temperature is mainly affected by the nucleation temperature of ice crystals in the mortar pores, the total void ratio, the pore size distribution, etc. It reflects the temperature at which the mortar pore solution starts to freeze in large quantities, and to a certain extent, it can characterize the change in the mortar pore structure. Comparing the characteristic temperatures T_F1_ and T_F100_ of D40-1% mortar after the 1st and 100th freeze–thaw cycles, the temperature increased from −5 °C to −4 °C, which to a certain extent reflects the development of internal defects in the mortar and the deterioration of the pore structure after 100 cycles of freezing–thawing, which is one of the reasons for the generation of the residual strain [27]. Notably, negligible residual strain was observed after the first freeze–-thaw cycle, whereas 18 με accumulated by the 100th cycle. This trend confirms that prolonged freeze–thaw cycling accelerates the deterioration of the mortar’s internal pore structure, leading to progressive residual strain accumulation.

**Figure 9:**
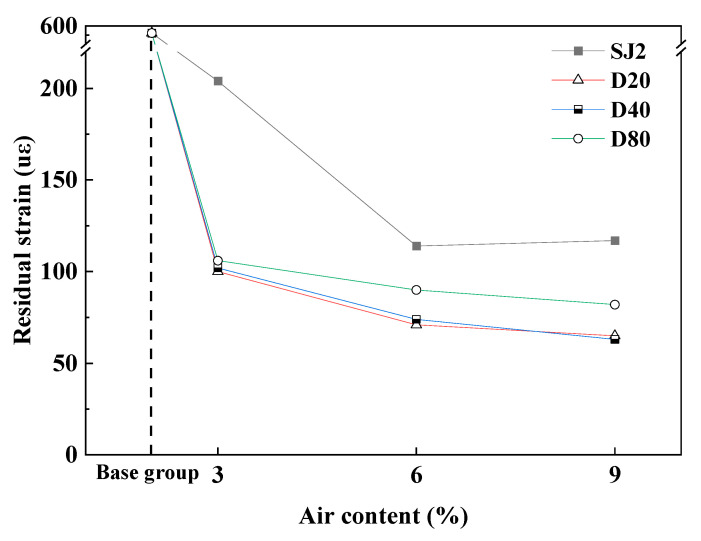
Residual strain after 100 freeze–thaw cycles.

In order to study the effects of different types of air-entraining, air content, and pore size on the residual strain of mortar after the freeze–thaw cycle, traditional air-entraining agent SJ2 mortar and three different particle size WEA air-entraining mortars were respectively selected to compare and analyze the residual strain after 100 freeze–thaw cycles, and the results are shown in Figure 9.

As can be seen in Figure 9, with the increase in gas content from 3% to 6%, the residual strain of each group of air-entraining mortar exhibits a large reduction; when the gas content increases from 6% to 9%, the residual strain of each group of air-entraining mortar slows down, and the residual strain of SJ2 air-entraining mortar increases slightly instead, which indicates that the excessive air-entraining is detrimental to the freezing performance of the material. It is worth noting that the residual strain of SJ2 air-entrained mortar is larger than that of WEA air-entrained mortar under the same air content because the air pores introduced by WEA are more homogeneous and regular, with higher spherical likelihood, and the spherical structure of the air pores can efficiently counteract the freezing and expansion stresses to improve the freezing performance [28]. Comparing the mortars with three different size intervals of introduced pores, it can be seen that the residual strain of the mortar increases with the increase in the size of pores.

The results demonstrate that the residual strain of the polymer-mixed hollow microsphere mortar after 100 freeze–thaw cycles is significantly lower than that of SJ2 air-entraining mortar. Furthermore, the pores introduced by WEA exhibit greater uniformity and regularity, enabling them to form a more stable pore structure that cushions and disperses freezing-induced expansion stresses, thereby enhancing the mortar’s frost resistance. For mortars with pores distributed across distinct size ranges, frost resistance follows the order of D20 > D40 > D80. Consequently, it is advisable to prioritize the introduction of favorable pores with smaller diameters in air-entrained concrete design while minimizing the incorporation of harmful large-diameter pores.

#### 3.4.2. Relative Dynamic Modulus of Elasticity and Spacing Factor

**Figure 10:**
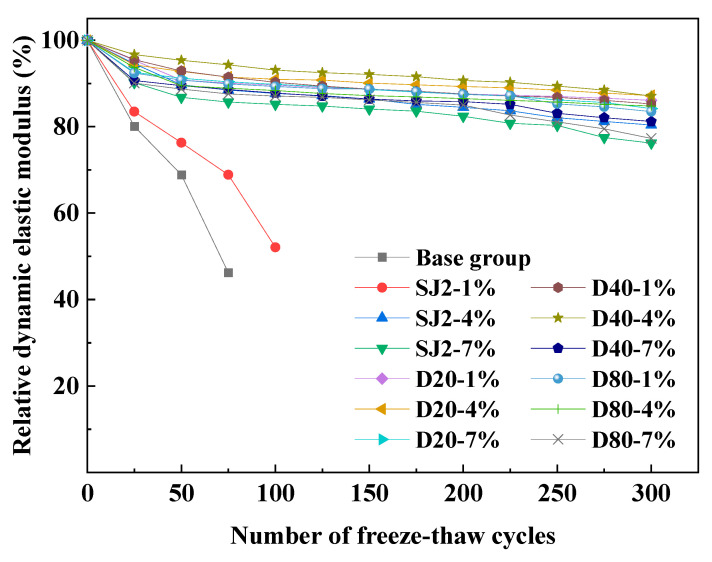
The relationship between the relative dynamic modulus of elasticity and the number of freeze–thaw cycles.

**Figure 11:**
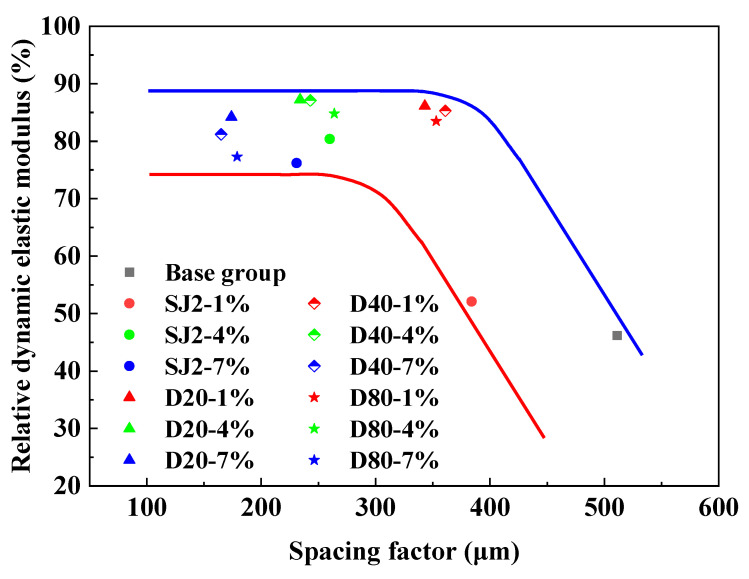
The relationship between the relative dynamic modulus of elasticity and spacing coefficient.

The relationship between the relative dynamic elastic modulus and the number of freeze–thaw cycles and spacing coefficient was analyzed using a freeze–thaw cycle test and hardened mortar pore structure test for each group of mortar, and the results are shown in Figure 10 and Figure 11. As can be seen in Figure 10, the relative dynamic elastic modulus of each group decreased to different degrees after 300 freeze–thaw cycle tests, among which the relative dynamic elastic modulus of the baseline group mortar and the SJ2-1% mortar dropped below 60%, and the tests were terminated after 75 and 100 freeze–thaw cycle tests, respectively. After the freeze–thaw cycle test, the relative dynamic elastic modulus of the benchmark mortar and SJ2-1% mortar decreased to less than 60%, and the test was terminated, and the other groups of air-entraining mortar were able to reach the frost resistance of F300. Overall, the frost resistance of WEA-entraining mortar was better than that of SJ2-entraining mortar, and the frost resistance of D40-1% mortar was comparable to that of SJ2-4%, in which the relative dynamic elastic modulus of D40-4% mortar was reduced by only 12.9%. As can be seen from Figure 11, the spacing coefficient of WEA-entrained mortar is smaller than that of SJ2-entrained mortar under the same air content. With the increase in gas content in each group of mortar, the spacing coefficient gradually decreased; with the introduction 7% gas content, the spacing coefficient of SJ2 air-entraining mortar did not change much compared with when the gas content was 4%, mainly because of the fusion of some of the small bubbles into the size of the larger bubbles, and the doping of the WEA introduced by the pores did not appear to merge the phenomena. When the spacing factor of SJ2 air-entraining mortar is less than 250 μm, it has freeze resistance, and when the spacing factor is 350 μm, the freeze resistance of SJ2 air-entraining mortar is better. When the spacing factor is 350 μm, the relative dynamic elastic modulus drops to less than 60% after 300 cycles of freezing and thawing, and the air-entraining mortar with a pore size of 20–80 μm introduced by polymer hollow microspheres still has good frost resistance with a spacing factor of about 350 μm.

Therefore, more regular and stable target-size pores can be introduced into the mortar with WEA to optimize the pore structure, avoiding the introduction of traditional air-entraining agents with pores of too large a size, so as to improve the frost resistance, and also to provide design ideas for the future air-entraining concrete pore gradation design of the new method.

## 4. Conclusions

In this study, the effect of polymer hollow microspheres (WEA, 20–80 μm) with a controllable particle size was investigated to determine its effect on the frost resistance of mortar, and a comparative study was carried out with a traditional air-entraining agent (SJ2). The differences in the pore structure and frost resistance of the two air-entraining mortars were systematically analyzed, and the following conclusions were obtained:

(1) Polymer hollow microspheres (WEA) consist of gas-filled cores encapsulated by a 100 nm thick thermoplastic shell, exhibiting superior mechanical properties and stability compared to the liquid film bubbles introduced by traditional SJ2 air-entraining agents (surfactants). The pore size introduced by WEA can be precisely controlled and stabilized, remaining unaffected by mortar molding or hydration processes, thereby forming uniformly sized, regularly shaped pores in the cement matrix.
(2) At equivalent air content, WEA-modified mortar demonstrates higher compressive strength and lower residual strain after 100 freeze–thaw cycles compared to SJ2-modified mortar, indicating that WEA significantly enhances both mechanical performance and frost resistance relative to SJ2.
(3) At a particle size of 40 µm, WEA-modified mortar with a 1% volume addition exhibits frost resistance comparable to that of SJ2-modified mortar with 4% air content. The performance of WEA-modified mortars is influenced by particle size, following the order of D20 > D40 > D80 in both mechanical strength and frost resistance.
(4) SJ2 air-entrained mortar exhibits superior frost resistance when the spacing coefficient is below 250 μm. However, when the spacing coefficient exceeds 350 μm, its relative dynamic elastic modulus drops below 60% after 300 freeze–thaw cycles. In contrast, WEA-modified mortar, with pore sizes ranging from 20 to 80 µm, maintains excellent frost resistance even at spacing coefficients around 350 µm.

In the subsequent research, the air-entraining effect of WEA air-entraining agents under different air pressure environments should be investigated, the pore structure changes of WEA air-entraining mortar under low air pressure environments should be analyzed, and the relationship between the pore structure parameters and frost resistance under low air pressure environments should be elucidated.

## Figures and Tables

**Table 1:** Chemical composition of cement (% in wt.).

SiO_2_	Al_2_O_3_	Fe_2_O_3_	CaO	MgO	SO_3_	Na_2_O	f-CaO	LOI
20.82	4.40	3.27	63.34	2.88	2.42	0.59	0.87	0.02

**Table 2:** Physical properties of cement.

Density (g·cm^−3^)	Specific Surface Area (m^2^ kg^−1^)	Condensation Time/Min		Flexural Strength/MPa			Compressive Strength/MPa		
		Condensation	Congeal	3 d	7 d	28 d	3 d	7 d	28 d
3.11	352	123	188	5.5	7.0	9.1	27.4	36.6	53.1

**Table 3:** Material parameters of WEA.

Norm	Particle Size (μm)	True Density (kg/m^3^)	Solid Content (%)
D20	20	36	15
D40	40	25	22
D80	80	45	25

**Table 4:** Mix proportion of the mortar.

WEA	Groups	Water/kg	Cement/kg	Sand/kg	Replacement Volume (%)	Dosage (g)	Target Gas Content (%)
D20	D20-1%	0.45	1.0	2.0	1	3.69	3
	D20-4%				4	14.76	6
	D20-7%				7	25.83	9
D40	D40-1%				1	1.75	3
	D40-4%				4	5.24	6
	D40-7%				7	12.23	9
D80	D80-1%				1	2.76	3
	D80-4%				4	8.32	6
	D80-7%				7	19.36	9

## Data Availability

The original contributions presented in this study are included in the article. Further inquiries can be directed to the corresponding authors.
